# Ecotyping of *Anaplasma phagocytophilum* from Wild Ungulates and Ticks Shows Circulation of Zoonotic Strains in Northeastern Italy

**DOI:** 10.3390/ani11020310

**Published:** 2021-01-26

**Authors:** Laura Grassi, Giovanni Franzo, Marco Martini, Alessandra Mondin, Rudi Cassini, Michele Drigo, Daniela Pasotto, Elena Vidorin, Maria Luisa Menandro

**Affiliations:** Department of Animal Medicine, Production and Health (MAPS), University of Padova, 35020 Legnaro, Padova, Italy; laura.grassi.2@phd.unipd.it (L.G.); giovanni.franzo@unipd.it (G.F.); marco.martini@unipd.it (M.M.); alessandra.mondin@unipd.it (A.M.); rudi.cassini@unipd.it (R.C.); michele.drigo@unipd.it (M.D.); daniela.pasotto@unipd.it (D.P.); elena.vidorin@gmail.com (E.V.)

**Keywords:** *Anaplasma phagocytophilum*, zoonosis, tick, wild ungulates, phylogenesis, molecular epidemiology

## Abstract

**Simple Summary:**

Tick-borne infectious diseases represent a rising threat both for human and animal health, since they are emerging worldwide. Among the bacterial infections, Anaplasma phagocytophilum has been largely neglected in Europe. Despite its diffusion in ticks and animals, the ecoepidemiology of its genetic variants is not well understood. The latest studies identify four ecotypes of Anaplasma phagocytophilum in Europe, and only ecotype I has shown zoonotic potential. The aim of the present study was to investigate the genetic variants of Anaplasma phagocytophilum in wild ungulates, the leading reservoir species, and in feeding ticks, the main vector of infection. The analyzed samples were collected in northeastern Italy, the same area where the first Italian human cases of anaplasmosis in the country were reported. Using biomolecular tools and phylogenetic analysis, ecotypes I and II were detected in both ticks (Ixodes ricinus species) and wild ungulates. Specifically, ecotype II was mainly detected in roe deer and related ticks; and ecotype I, the potentially zoonotic variant, was detected in Ixodes ricinus ticks and also in roe deer, red deer, chamois, mouflon, and wild boar. These findings reveal not only the wide diffusion of Anaplasma phagocytophilum, but also the presence of zoonotic variants.

**Abstract:**

*Anaplasma phagocytophilum* (*A. phagocytophilum*) is a tick-borne pathogen causing disease in both humans and animals. Human granulocytic anaplasmosis (HGA) is an emerging disease, but despite the remarkable prevalence in European ticks and wild animals, human infection appears underdiagnosed. Several genetic variants are circulating in Europe, including the zoonotic ecotype I. This study investigated *A. phagocytophilum* occurrence in wild ungulates and their ectoparasites in an area where HGA has been reported. Blood samples from wild ungulates and ectoparasites were screened by biomolecular methods targeting the *mps2* gene. The *groEL* gene was amplified and sequenced to perform genetic characterization and phylogenetic analysis. A total of 188 blood samples were collected from different wild ungulates species showing an overall prevalence of 63.8% (88.7% in wild ruminants and 3.6% in wild boars). The prevalence of *A. phagocytophilum* DNA in ticks (manly *Ixodes ricinus*), and keds collected from wild ruminants was high, reflecting the high infection rates obtained in their hosts. Among ticks collected from wild boars (*Hyalomma marginatum* and *Dermacentor marginatus*) no DNA was detected. Phylogenetic analysis demonstrated the presence of ecotype I and II. To date, this is the first Italian report of ecotype I in alpine chamois, mouflon, and wild boar species. These findings suggest their role in HGA epidemiology, and the high prevalence detected in this study highlights that this human tick-borne disease deserves further attention.

## 1. Introduction

*Anaplasma phagocytophilum* (*A. phagocytophilum*) is a small, Gram-negative, obligate intracellular bacterium belonging to the Anaplasmataceae family, order Rickettsiales [1]. It infects white blood cells, mainly neutrophilic granulocytes, and is the causal agent of human granulocytic anaplasmosis (HGA), tick-borne fever (TBF), or pasture fever of domestic ruminants and granulocytic anaplasmosis of horses (equine granulocytic anaplasmosis, EGA), dogs (canine granulocytic anaplasmosis, CGA) and cats (feline anaplasmosis, FA) [2,3].

*Anaplasma phagocytophilum* is a tick-borne pathogen mainly transmitted, in Europe, by the *Ixodes* genus, especially *I. ricinus*. This bacterium has been detected in other hard tick species, but their vectorial efficiency seems negligible [3,4,5]. Occasionally, HGA can be caused by blood transfusion, transplacental transmission, or direct contact with infected blood of wild ungulates at butchering [6]. However, tick bites represent the main route of transmission, and once infection is established, ticks maintain *A. phagocytophilum* through trans-stadial transmission, while the transovarial route seems not to be efficient [7]. Accordingly, mainly nymphal and adult stages are involved in bacterial transmission [8]. *Ixodes ricinus* is therefore considered an efficient vector but not a reservoir [4], thus vertebrate species, especially wildlife, seem to be pivotal in maintaining *A. phagocytophilum* circulation [9,10].

Prevalence in *I. ricinus* ticks is reported to increase according to the number of blood meals. Adult ticks therefore show higher infection rates than nymphs and larvae [4], reaching even 100% in adult stages collected from wild ungulates [11]. Wild ungulates harbor relevant amounts of all tick stages [11] and play a critical role in the transmission cycle of anaplasma, as they act as both tick hosts and reservoir species of *A. phagocytophilum* [12]. In Europe, roe deer (*Capreolus capreolus*) and red deer (*Cervus elaphus*) in particular show very high *A. phagocytophilum* prevalence [4]. Although other wild ruminants may display a non-negligible infection frequency, their role has not yet been established [8]. In wild boar, *A. phagocytophilum* infection is less common, but this ungulate has been suggested as a reservoir of zoonotic strains [11,13]. Indeed, the genetic diversity of *A. phagocytophilum* strains has been demonstrated, and the genetic variants seem to display a host preference, showing different epidemiological and clinical pictures [8,10]. The genes commonly used to genotype and distinguish these variants are 16S *rRNA*, *groEL*, *ankA*, and *msp4*. Among these, the *groEL* gene, belonging to *groESL* heat shock operon, seems to be a good marker of *A. phagocytophilum* genetic diversity. It is mostly studied in Europe, where the genetic variability is greater than that observed in other geographical areas, such as North America [3,10].

Based on the *groEL* classification, four ecotypes, subdivided into eight clusters, have been identified in different tick and vertebrate species [10,14]. Ecotype I has the widest host range, and is found in livestock, dogs, cats, horses, and wild species, including carnivores, small mammals, wild boars, and other ungulates. Also, human infections are driven by ecotype I, which is of primary importance in the epidemiology of HGA [3,14]. Other ecotypes seem to be preferentially associated with certain species. Ecotype II is detected mainly in roe deer and only sporadically in other species, ecotype III is linked to small mammals, especially rodents, and ecotype IV is found mainly in birds. This distribution seems to also be related to the host preference of different species of ticks. Ecotypes I and II are mainly detected in *I. ricinus* [10]. Both ecotypes have also been identified in other ectoparasites such as deer keds (*Lipoptena cervi*, *L. cervi*) [14]. The role of this species in the transmission of anaplasmosis has been suggested but not demonstrated [15]. Ecotypes III and IV have been detected in *Ixodes trianguliceps* and *Ixodes frontalis*, which usually feed on rodents and birds, respectively [14]. The distribution of European ecotypes thus seems to be affected by vertebrate host and vector species, their interaction and, consequently, geographic origin [3,10]. Although ecotypes seem more represented in some preferential animal species, many studies show their overlap within the same mammal species, suggesting that *A. phagocytophilum* variants may not be host-specific at all [3,16].

Human granulocytic anaplasmosis has been reported worldwide, but is mainly detected in the northern hemisphere [17]. The first human cases in the USA and Europe were identified in 1990 and 1996, respectively [18,19]. In Italy, the first HGA cases were reported in the Friuli–Venezia–Giulia region in 2000 [20], followed by other cases reported in Sicily and Sardinia [21,22]. Despite high infection rates of *A. phagocytophilum* in ticks and in different wild and domestic species, HGA seems to be rare at present. In Italy, 16 different tick species have been reported to bite humans, in Northeastern Italy, almost all cases are attributed to *I. ricinus* [23,24,25]. A possible reason for the low HGA prevalence in Italy is that it is not a notifiable disease, which could lead to underestimation of disease incidence [24]. In addition, the subclinical or poorly specific nature of *A. phagocytophilum*-induced symptoms further decreases the recognition and reporting infections. However, if not treated, it could evolve into more severe and even fatal syndromes. Immunocompromised patients are at higher risk of high morbidity and mortality [26]. Based on these findings, many authors agree that HGA is neglected and underdiagnosed, and thus underestimated [3,9,26].

Although the studied area, northeastern Italy, is the same place where the first Italian cases of HGA were reported [20,27], to date, there are few epidemiological and no phylogenetic studies on this pathogen in wild ungulates, which may act as an important reservoir of *A. phagocytophilum* variants related to HGA. Thus, this study aims to fill this gap, evaluating the presence and distribution of zoonotic *A. phagocytophilum* ecotypes in wildlife and in their associated ectoparasites.

## 2. Materials and Methods

### 2.1. Sampling Sites and Specimens Collection

The area under investigation comprises several different sites in northeastern Italy. The first comprises pre-alpine and alpine localities in the province of Udine in the Friuli–Venezia–Giulia region. The other two, both located in the Veneto region, included pre-alpine and alpine areas in Belluno province, and hilly areas in Euganean Hills Regional Park. The highest mountain peak of alpine areas reaches an elevation of 2500 m above sea level (a.s.l.), while Euganean hills are characterized by a maximum elevation of 600 m a.s.l. The hunting reserves are all characterized by the presence of wild ungulate populations, that increased remarkably in the last decades as a consequence of animal protection and reintroduction policies or illegal release of gaming animals [28]. In particular, free-ranging wild ruminants are particularly present in pre-alpine and alpine areas, where the most abundant are roe deer and red deer, followed by chamois and mouflon; these areas are also colonized by free-ranging wild boar populations [29,30]. In contrast, only free-ranging wild boars and fallow deer are present in the hilly areas of Euganean Hills Regional Park [31].

Blood and ectoparasites were collected from culled wild ungulates by hunters during the hunting seasons of 2017–2018 and 2018–2019, lasting from spring to winter. Hunters were instructed and trained on sampling procedures and data collection, reporting the features of killed animals (species, sex, and estimated age), and the place and the date of hunting. To avoid cross-contamination linked to field conditions, a selection of samples was carried out, i.e., collected when only one animal was killed per day for each hunter. Whole-blood samples were retrieved by cutting the jugular vein or main vessels of the chest cavity and collected in 9 mL Vacumed^®^ with K3EDTA tubes (FL Medical s.r.l., Torreglia, Padova, Italy), refrigerated as soon as possible, and brought to the veterinary infectious disease laboratory (Department of Animal Medicine, Production and Health, Padua University, Italy) within 4 days. The blood samples were divided into 200 μL aliquots and stored at −80 °C until analysis.

When present, the matching ectoparasites were also collected before animal exsanguination and brought to the laboratory in sterile plastic tubes. The identification of tick species was performed with stereomicroscope and microscope, following the identification keys described by Cringoli et al., then the parasites were stored at −80 °C until analysis [32].

A total of 188 blood samples were collected from wild ungulates: 17 from Euganean Hills Regional Park, 62 from an alpine area of the Veneto region, and 109 from an alpine area of the Friuli– Venezia–Giulia region. Six species were sampled: Alpine chamois (*Rupicapra rupicapra*; n = 9), roe deer (*Capreolus capreolus*; n = 74), red deer (*Cervus elaphus*; n = 39), mouflon (*Ovis musimon*; n = 8), fallow deer (*Dama dama*; n = 3), and wild boar (*Sus scrofa*; n = 55). A subsample of ticks (up to a maximum of 10) was collected from all infested ungulates. With the exception of alpine chamois, wild ruminant species had a higher tick burden compared to wild boar. In total, 277 ticks were collected and analyzed. The following tick species were identified: *I. ricinus* (n = 258), *Dermacentor marginatus* (*D. marginatus*) (n = 18) and *Hyalomma marginatum* (*H. marginatum*) (n = 1). In addition, also 15 deer keds (*L. cervi*) were found. When more than one tick was collected from the same host, ticks were analyzed in pools composed of a maximum of 2 specimens characterized by the same species, gender, and stage. Thus, a total of 216 samples were tested for *A. phagocytophilum*: 201 tick samples (pooled ticks = 76; individual ticks = 125) and 15 deer keds.

### 2.2. Biomolecular Analysis

DNA was extracted from 200 μL of whole blood and from tick homogenates using the DNeasy Blood & Tissue Kit (QIAGEN, Hilden, Germany), according to the manufacturer’s instructions. A canine blood sample, certified as negative to *A. phagocytophilum* infection by the Italian authority and research organization for animal health and food safety (Istituto Zooprofilattico delle Venezie, IZSVe), was included in each extraction run as an extraction negative control and thereafter tested together with the diagnostic samples. Prior to extraction, an exogenous DNA internal control (supplied by QuantiNova Pathogen + IC kit, QIAGEN) was spiked in all blood and ectoparasites specimens. To exclude false negative results due to the presence of polymerase inhibitors, all DNA samples were checked in duplicate for the presence of the internal control DNA, according to the manufacturer’s instructions. DNA samples showing no inhibition were then screened for the presence of *A. phagocytophilum* using real-time PCR targeting a 77 bp portion of the *msp2* gene (Table 1) [33].

All real-time PCR reactions were performed on a LightCycler96 instrument (Roche, Basel, Switzerland) using QuantiNova Pathogen + IC Kit reagents (QIAGEN, Hilden, Germany) in a final reaction volume of 7 μL, consisting of 0.8 μM of each primer, 0.25 μM of probe and 2 µL of DNA sample. The following thermal protocol was used: Preincubation at 95 °C for 120 s, followed by 50 cycles of denaturation at 95 °C for 5 s, annealing at 60 °C for 5 s, and extension at 72 °C for 30 s. Internal, positive, and negative controls were included in each run. Samples were considered positive if both replicates showed an amplification curve with a mean Cq value lower to 40.00 or equal.

To genetically characterize *A. phagocytophilum* strains, positive samples were tested by conventional PCR using primers targeting a 600 bp portion of *groEL* gene (Table 1) [34]. As a target, 5 µL of DNA was used in a 25 µL reaction mixture containing 0.3 µM of each primer and 1× Phire Hot Start II PCR Master Mix (Thermo Fischer Scientific, Milano, Italy). Amplifications were carried out in a TGradient thermal cycler (Biometra, Analytic Jena GmbH, Jena, Germany) in the following conditions: Initial denaturation at 98 °C for 60 s, followed by 50 cycles of denaturation at 98 °C for 5 s, annealing at 60 °C for 5 s, and extension at 72 °C for 7 s. Negative and positive controls were included in each run. PCR products were visualized by 2% agarose gel electrophoresis containing SybrSafe DNA (Thermo Fischer Scientific, Milano, Italy) gel stain in Tris-borate-EDTA (TBE) buffer (Merk KGaA, Darmstadt, Germany) using GelDoc EZ Imager (Bio–Rad Laboratories, Milano, Italy). Amplicons were enzymatically purified with ExoSap-IT Express PCR Product Cleanup (Thermo Fischer Scientific, Milano, Italy) and delivered to an external laboratory (StarSEQ^®^, Mainz, Germany) to be sequenced in both directions using the same PCR primers.

### 2.3. Statistical Analysis

Logistic regression models were fitted to evaluate the association between *A. phagocytophilum* infection and host features. The following categorical variables were considered in univariable and multivariable models: Area (Euganean Hills Regional Park, Friuli–Venezia–Giulia Alps, Veneto Alps), season (spring, summer, autumn, winter), gender (female, male), age (<1 year, ≥1 year), and species (chamois, red deer, roe deer, wild boar). Due to the small number of tested individuals, fallow deer and mouflon were not included among the considered species. The analysis was performed also considering the variable species as dichotomous (ruminant, wild boar), also including in that case fallow deer and mouflons. A model was preferred over a simpler one when a significant improvement on the overall fit was demonstrated by likelihood ratio test. All analyses were performed in SPSS v23.0 (IBM Corporation, Armonk, NY, USA): The significance level was set to *p* < 0.05.

### 2.4. Sequence Analysis

Consensus sequences were generated using ChromasPro Software v.2.1.8 (Technelysium Pty Ltd., South Brisbane, QLD, Australia) and compared with representative sequences available in the National Center for Biotechnology GenBank database with the BLASTn tool [35]. The genetic analysis was performed according to the classification proposed by Jahafari et al. and implemented by Jaarsma et al., based on *groEL* gene sequencing [10,14].

DNA sequences corresponding to the region between nucleotide positions 533 and 1033 of the CP015376 *groEL* open reading frame obtained in the present study, were aligned to 1998 sequences representative of the 4 ecotypes and 8 clusters previously described [10] using MAFFT v7.450 [36]. Additionally, the Italian sequences reported by Di Domenico et al., were also included in the study [37]. To reduce the tree complexity and computational burden, only one sequence representative of all identical ones was identified using CD-HIT and included in the phylogenetic tree (Table S1) [38]. The sequence suitability for phylogenetic analysis was assessed by likelihood mapping analysis, performed using IQ-TREE, and a phylogenetic tree was reconstructed with the same software, selecting as a substitution model the one with the lowest Akaike information criterion (AIC), value calculated using JmodelTest [39,40]. The reliability of inferred clades was investigated by performing 10,000 ultrafast bootstrap replicates.

## 3. Results

### 3.1. Wild Ungulates

The overall prevalence of *A. phagocytophilum* in wild ungulates was 63.8% (95% CI: 56.7–70.3%; 120 out of 188). However, the prevalence was above 75% in all ruminant species and significantly higher (overall ruminants prevalence = 88.7% (95% CI: 83.4–94.1; 118 out of 133) than that observed in wild boar (3.6%; 95% CI: 0–8.6; 2 out of 55). Wild ruminants thus have 208 times higher odds of being infected (95% CI: 57.1–504.1; *p* < 0.001) (Table 2). No significant differences were identified among wild ruminant species (*p* = 0.454).

Other statistically significant differences in *A. phagocytophilum* infection prevalence were observed in univariable analysis among the three different areas. Particularly, the odds ratios were 8.4 (95% CI: 2.5–37.9; *p* = 0.001) and 14.6 (95% CI: 4.1–69.9; *p* < 0.001) higher in Friuli–Venezia–Giulia and the Veneto Alps than in the Euganean Hills Regional Park, respectively. These differences should be evaluated considering the species distribution, which can act as confounder. In particular, wild boar samples were collected especially from the Euganean Hills Regional Park during spring. In fact, taking into account host distribution in the multivariable analysis, the differences among geographic areas became non-significant. A significant difference was observed among seasons, with summer, autumn, and winter showing odds of 4.7 (95% CI: 1.8–12.8; *p* = 0.001), 5.6 (95% CI: 2.2–15.7; *p* < 0.001) and 4.0 (95% CI: 1.1–17.2; *p* = 0.046) higher than spring. Also in this case, seasonal patterns of wild species culling can be considered a confounder, and no significant difference in seasonality was observed when the host species was included in the multivariable model. No significant differences were observed according to gender (*p* = 0.871) or age (*p* = 0.639).

### 3.2. Ectoparasites

Out of 201 tick samples, 122 were *I. ricinus* adult ticks, mainly female (142/182); adult male (38/182) and nymphal stages (2/182) were less frequent. All *D. marginatus* (10/18 females and 8/18 males) and *H. marginatum* (1 male) were collected from wild boars at Euganean Hills Regional Park. *L. cervi* keds were found on roe deer (9/15), red deer (3/15), and wild boars (3/15).

Real-time PCR positive results were obtained in 63.7% of ticks (95% CI: 56.5–70.2; 128 out of 201) of ticks and in 40.0% of deer keds (95% CI: 17.4–67.1; 6 out of 15). Bacterial DNA was not detected in *D. marginatus* and *H. marginatum* that were collected from negative wild boars. One out of two nymphal pools tested positive.

Results of real-time PCR analysis of tick *A. phagocytophilum* were grouped according to several variables (Table 3). Twelve host–parasite pairs displayed at least one missing feature (e.g., sex, engorgement, etc.). Therefore, a total of 189 records were included in this final analysis. Tick positivity to *Anaplasma* infection was differently distributed according to host species, host infectious status, and tick sex (Table 3).

### 3.3. Ecotypes and Clusters

**Figure 1:**
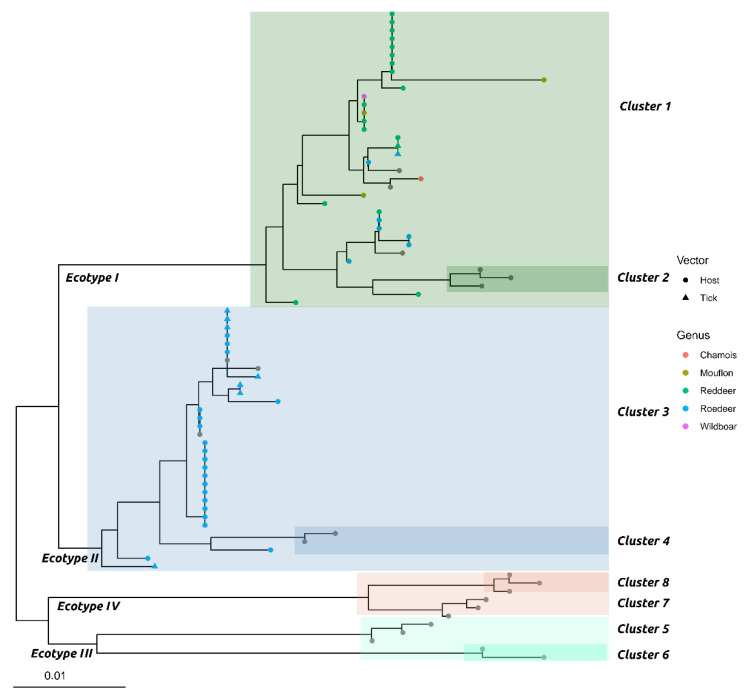
Maximum likelihood phylogenetic tree based on *groEL* gene of Italian strains collected from wild ungulates (dots) and their *Ixodes ricinus* ticks (triangles). Species of origin are color-coded. Ecotypes and respective clusters are highlighted with different colors. For graphical reasons, only a subset of reference sequences [10] is included.

Only samples positive with real-time PCR screening (120 blood samples, 128 tick samples, and 6 keds) were further analyzed by conventional PCR, targeting a portion of the *groEL* gene to ecotype them. Fifty-seven good quality sequences were obtained when mean Cq values between the two replicates in real-time PCR were below 33.00. Of these 57 sequences, 48 were from blood samples and 9 from *I. ricinus* feeding ticks (GenBank accession numbers from MT473440 to MT 473496) and were used for bioinformatics analysis (Figure 1 and Figure S1 and Table S2).

Two *A. phagocytophilum* from ticks and 28 from animals were classified as ecotype I, cluster 1 (n = 30). These were derived from wild boar (n = 1), alpine chamois (n = 1), mouflon (n = 3), roe deer (n = 6), and red deer (n = 17). The ticks infected with ecotype I were hosted by one roe deer and one red deer. All other *A. phagocytophilum* sequences (n = 27) were classified as ecotype 2, and all were grouped in cluster 3, sampled from roe deer (n = 20) and related ticks (n = 7). Of note, a tick infected with *A. phagocytophilum* belonging to ecotype I was feeding on an ecotype II positive roe deer. Further details are shown in Supplementary Files (Figure S1 and Table S2).

## 4. Discussion

In European countries *A. phagocytophilum* infection has been reported in a wide range of wild animals, including several species of rodents, hares, wild carnivores, birds, reptiles, and especially wild ungulates species [4,8]. This research investigated the epidemiology and phylogenesis of *A. phagocytophilum* infection in wild ungulates and related ectoparasites. We report widespread circulation of *A. phagocytophilum* in sylvatic cycles in northeastern Italy.

The overall *A. phagocytophilum* prevalence detected in wild ungulates was 63.8%. A high prevalence was reported in roe deer (91.9%) and red deer (87.2%), irrespective of the age (Table 2). This evidence supports their reservoir role [16,41]. Previous Italian studies reported both similar [37,42,43] and much lower prevalence data in wild ungulates [44,45,46,47,48]. *A. phagocytophilum* was also frequently detected in the other wild ruminant species, i.e., mouflon (75%), chamois (77.8%), and fallow deer (100%). Although only a limited number of samples of these species were analyzed in the present study, which were not representative of the target population, our data are in line with other studies [12,37,49], suggesting that more attention should be given to their impact on the anaplasmosis infectious cycle. An opposite role emerged for wild boars, with a much lower prevalence (3.6%) observed. This finding seems related to the lower quantity of ticks harbored by wild boars [11,50], thus decreasing the probability of wild boar infection.

No statistical association has been observed regarding the host prevalence and sex, age and, interestingly, seasonality. Actually, ticks have been collected from wild ruminants throughout the year, at similar frequency. This finding could be due to an increased tick survival and activity even during the winter months because of global warming [51,52].

Our research confirms the pivotal role of *I. ricinus* as the main vector of *A. phagocytophilum*, compared to other hard tick species, as previously reported [8,53]. The high prevalence (63.7%) of *A. phagocytophilum* detected in ticks collected from wild ungulates in northeastern is related to the reservoir role of these animals and the engorgement status of ticks [11]. Previous studies on *I. ricinus* removed from wild ungulates in other Italian regions, however, reported a lower prevalence, ranging from 5.1 to 31.2% [37,44,54,55,56], even when the same analytical methods was used. This evidence highlights that the prevalence of this bacterium depends not only on the presence of *I. ricinus* and wild ungulates, but on complex interactions involving the entire ecosystem, resulting in different enzootic cycles depending on the studied area, even within the same country [14,57]. Twelve positive ticks were collected from negative animals (Table 3) and three were non-engorged males. Therefore, infection may have been acquired in a previous stages and maintained through trans-stadial transmission, as previously reported by other authors [3,14]. However, the higher percentage of *A. phagocytophilum* infected ticks found on positive wild ungulates confirms the efficient transmission between these reservoir species and the vector. In this study mainly adult ticks were collected from ungulates, but larvae and nymphs can also parasitize these animals and some of them could maintain the infection through trans-stadial transmission [11]. Previous studies conducted on questing ticks in the same study area showed a percentage of *A. phagocytophilum* infection up to 9% in *I. ricinus* ticks from Belluno province [58,59] and up to 9.9% in ticks from a neighboring area [60]. A lower prevalence (1.5%) was found when a more extensive territory, including sites near the plain, was investigated [61]. On the other hand, the lack of *A. phagocytophilum* detection in *Dermacentor* and *Hyalomma* ticks supports a secondary or negligible role in anaplasmosis transmission [3,8]. Alternatively, they could be involved in the anaplasma infectious cycle only in areas where *I. ricinus* populations are particularly rare or absent, which is not the case of the considered region [5,57]. Moreover, *Dermacentor* and *Hyalomma* ticks have been found only on wild boars in the Euganean Hills Regional Park, an area characterized by a maximum altitude of 600 m a.s.l. where wild boars are more represented than ruminants, consisting mainly of fallow deer. Ecological conditions thus may be poorly comparable with other wilder areas where several wild ruminant populations share the same habitat and where mainly *I. ricinus* is detected. Therefore, a potential biasing effect of the host species and ecological niche cannot be excluded.

Of interest is the 40% *A. phagocytophilum* positivity in *L. cervi*. These ectoparasites were collected from positive red and roe deer. Although their role in the anaplasmosis infectious cycle is still under debate, other authors report deer keds as potential carriers of *A. phagocytophilum*, raising questions about their possible vectorial capability [15,62].

The genetic analysis of the 57 sequences performed, following the classification proposed by Jahafari et al. and Jaarsma et al., showed different distributions of ecotypes and clusters [10,14]. Of the 26 sequences from roe deer, 20 belonged to ecotype II/cluster 3. Ecotype II thus seems strictly related to this species [14,16]. On the other hand, ecotype I/cluster 1 showed the widest host range, having been found in roe deer (6/26), red deer (17/17), chamois (1/1), mouflon (3/3), and wild boar (1/1) species. Interestingly, the same clade includes genetic sequences of *A. phagocytophilum* strains from human cases in Europe; sequences MT473492, MT473484, MT473481, MT473458, MT473457, MT473453, MT473452, and MT473443 from red deer and sequence MT473485 from roe deer obtained in the present study were genetically identical to strains previously sampled from humans in Belgium (BelgiumPatient2014) and the Netherlands (LT06052), respectively (Table S1). Our finding suggests that these species could be a reservoir for zoonotic *A. phagocytophilum* variants. Even if an intense debate is still going on among authors on the actual role of red deer in ecotype I ecology and zoonotic risk [16], the high prevalence and association with this ecotype observed in our study favors their relevance and should thus be considered when evaluating the epidemiological risk for HGA [9,11,14]. The detection of ecotype I in wild boar highlights that it might be involved in anaplasmosis epidemiology, with the ability to harbor zoonotic variants [13,63]. However, the role of this species in anaplasmosis epidemiology needs further investigation, as it quickly eliminates *A. phagocytophylum* infection and has a lower tick burden [8].

Although chamois and mouflon were not considered to be pivotal in anaplasmosis diffusion, the remarkable prevalence herein reported and the demonstration that they can harbor ecotype I deserve more attention. To the best of the authors’ knowledge, this is the first Italian report of ecotype I in wild boar, alpine chamois, and mouflon.

Most of the sequences obtained from *I. ricinus* ticks belonged to ecotype II/cluster 3 (7/9) and were collected from roe deer, while the remaining (2/9) were associated with ecotype I/cluster 1. In detail, one of these two ecotype I positive ticks was collected from a red deer and, interestingly, the other one was collected from a roe deer that tested positive to ecotype II, suggesting the potential simultaneous presence of different *A. phagocytophilum* variants.

In line with these results, some authors reported that *I. ricinus* ticks do not play a role in the host specificity of *A. phagocytophilum* variants, as it has been demonstrated that they may harbor all *A. phagocytophilum* ecotypes [10]. Furthermore, the generalist and teletropic feeding behavior of *I. ricinus* could facilitate the continuous exchange of ecotypes, and this behavior could explain the wide diffusion of ecotype I in almost all tested ungulates [5]. At the same time, our findings support that host specificity appears to be driven mainly by the variants themselves rather than tick feeding habits and host infection susceptibility. The presence of an overlap in ecotype host distribution further complicates the estimation of circulating ecotypes. In the present study, this emerged clearly in roe deer in which both ecotypes have been detected, accordingly to the findings of Remesar et al. [64]. Since the simultaneous presence of more than one variant can occur [65], it cannot be under-emphasized that the routinely applied Sanger sequencing did not allow identification of multiple strains, just the most abundant one, which may have resulted in an underreporting of such co-infections.

The findings of this study highlight the concrete risk of humans acquiring *A. phagocytophilum* infection from tick bites in the investigated area. Moreover, other transmission routes should be also considered. Contact with animal infected blood has been suggested as a potential infection source, particularly for people manipulating carcasses of ungulates or their meat [6]. Because of the subclinical infection in immunocompetent people, HGA should also be taken in account in blood transfusions. Unfortunately, currently HGA occurrence has rarely been investigated, especially through adequate genetic characterization, and further studies will be necessary to estimate its actual prevalence and relevance.

## 5. Conclusions

*A. phagocytophilum* was detected in all tested wild ungulates, with a higher prevalence in wild ruminants. Wild ungulates are involved in the *A. phagocytophilum* infectious cycle as the pivotal hosts for *I. ricinus* development and reproduction, harboring different *A. phagocytophilum* genetic variants. Of the detected variants, ecotype II/cluster 3 showed a host specificity for roe deer, while a broader tropism and the presence of overlapping niches emerged for ecotype I. Some sequences of ecotype I/cluster 1 from red deer and roe deer were identical to sequences from European human cases, highlighting their zoonotic potential. HGA itself may not cause severe disease, but it has been detected in co-infection cases with other tick-borne pathogens, leading to more severe symptomatology or atypical clinical presentations [27,66]. Due to the presence of zoonotic variants in the investigated area, the risk of infection should be communicated to categories of people at risk, such as hunters, veterinarians, and forest rangers, among others. Moreover, the often neglected HGA deserves more attention and should be considered in the differential diagnosis of human tick-borne diseases.

## Figures and Tables

**Table 1:** List of primers and probes used for *Anaplasma phagocytophilum* detection and sequencing.

Biomolecular Method	Target Gene	Primer	Nucleotide Sequence 5′–3′	Reference
Real-time PCR	*msp2*	ApMSP2f	ATGGAAGGTAGTGTTGGTTATGGTATT	[33]
		ApMSP2r	TTGGTCTTGAAGCGCTCGTA	-
		ApMSP2p	HEX-TGGTGCCAGGGTTGAGCTTGAGATTG-BHQ1	-
PCR	*groEL*	groEL643f	ACTGATGGTATGCARTTTGAYCG	[34]
		groEL1236r	TCTTTRCGTTCYTTMACYTCAACTTC	-

**Table 2:** Prevalence of *Anaplasma phagocytophilum* detected by real-time PCR in ungulate species grouped according to different variables. EHR, Euganean Hills Regional Park; FVG, Friuli–Venezia–Giulia.

Sample Features		Roe Deer Pos/N Prevalence	Red Deer Pos/N Prevalence	Mouflon Pos/N Prevalence	Alpine Chamois Pos/N Prevalence	Fallow Deer Pos/N Prevalence	Wild Boar Pos/N Prevalence
Ungulate		68/74 (91.9%)	34/39 (87.2%)	6/8 (75.0%)	7/9 (77.8%)	3/3 (100%)	2/55 (3.6%)
Gender	Female	22/24 (91.7%)	14/15 (93.3%)	3/3 (100%)	1/2 (50.0%)	1/1 (100%)	1/20 (5%)
	Male	46/50 (92%)	20/24 (83.3%)	3/5 (60.0%)	6/7 (85.71%)	2/2 (100%)	1/35 (2.9%)
Age *	<1 year	25/26 (96.2%)	10/11 (90.9%)	2/2 (100%)	2/2 (100%)	1/1 (100%)	1/25 (4.0%)
	>1 year	41/46 (89.1%)	23/27 (85.2%)	4/6 (66.7%)	5/7 (71.4%)	2/2 (100%)	0/28 (0%)
Season	Spring	6/7 (85.71%)	1/1 (100%)	-	-	-	1/18 (5.6%)
	Summer	38/42 (90.5%)	8/9 (88.9%)	1/1 (100%)	2/3 (66.7%)	1/1 (100%)	0/18 (0%)
	Autumn	20/21 (95.2%)	22/26 (84.6%)	5/7 (71.4%)	4/5 (80.0%)	2/2 (100%)	0/13 (0%)
	Winter	4/4 (100%)	3/3 (100%)	-	1/1 (100%)	-	1/6 (16.7%)
Area	EHR Park	-	-	-	-	3/3 (100%)	0/14 (0%)
	Veneto Alps	23/24 (95.8%)	19/22 (86.4%)	3/4 (75.0%)	-	-	2/12 (16.7%)
	FVG Alps	45/50 (90%)	1/17 (5.9%)	3/4 (75.0%)	7/9 (77.8%)	-	0/29 (0%)

* Ages of four positive animals (2 roe deer, 1 red deer, and 1 wild boar) and one negative wild boar were not available.

**Table 3:** Real-time PCR positivity of *Anaplasma phagocytophilum* in ticks according to different host and vector variables. Only records with complete data for both hosts and ectoparasites are included.

Sample Features		Tick Infection: Neg. (N = 69)	Tick Infection: Pos. (N = 120)
Host Species	Roe deer	35/69 (50.7%)	75/120 (62.5%)
	Red deer	5/69 (7.2%)	37/120 (30.8%)
	Mouflon	4/69 (5.8%)	6/120 (5.0%)
	Alpine chamois	1/69 (1.4%)	0/120 (0.0%)
	Fallow deer	2/69 (2.9%)	2/120 (1.7%)
	Wild boar	22/69 (31.9%)	0/120 (0.0%)
Host Infection	Neg.	28/69 (40.6%)	12/120 (10.0%)
	Pos.	41/69 (59.4%)	108/120 (90.0%)
Tick Sex	Female	43/69 (62.3%)	102/120 (85.0%)
	Male	26/69 (37.7%)	18/120 (15.0%)

## Data Availability

The data presented in this study are available in the text and in Table 2 and Table 3 and Figure 1 of this article and in the Figure S1, Tables S1 and S2.

## Supplementary Materials

The following are available online at https://www.mdpi.com/2076-2615/11/2/310/s1, Figure S1: Phylogenetic tree of *Anaplasma phagocytophilum* strains. Table S1: List of representative sequence used for phylogenetic tree. Table S2: Accession numbers of new Italian *Anaplasma phagocytophilum* sequences deposited into GeneBank.
