# Supplementary figures and images for: Ecotyping of Anaplasma phagocytophilum from Wild Ungulates and Ticks Shows Circulation of Zoonotic Strains in Northeastern Italy
Source: Animals (Basel). 2021 Jan 26;11(2):310. doi: 10.3390/ani11020310 (PMC7911980; doi:10.3390/ani11020310)

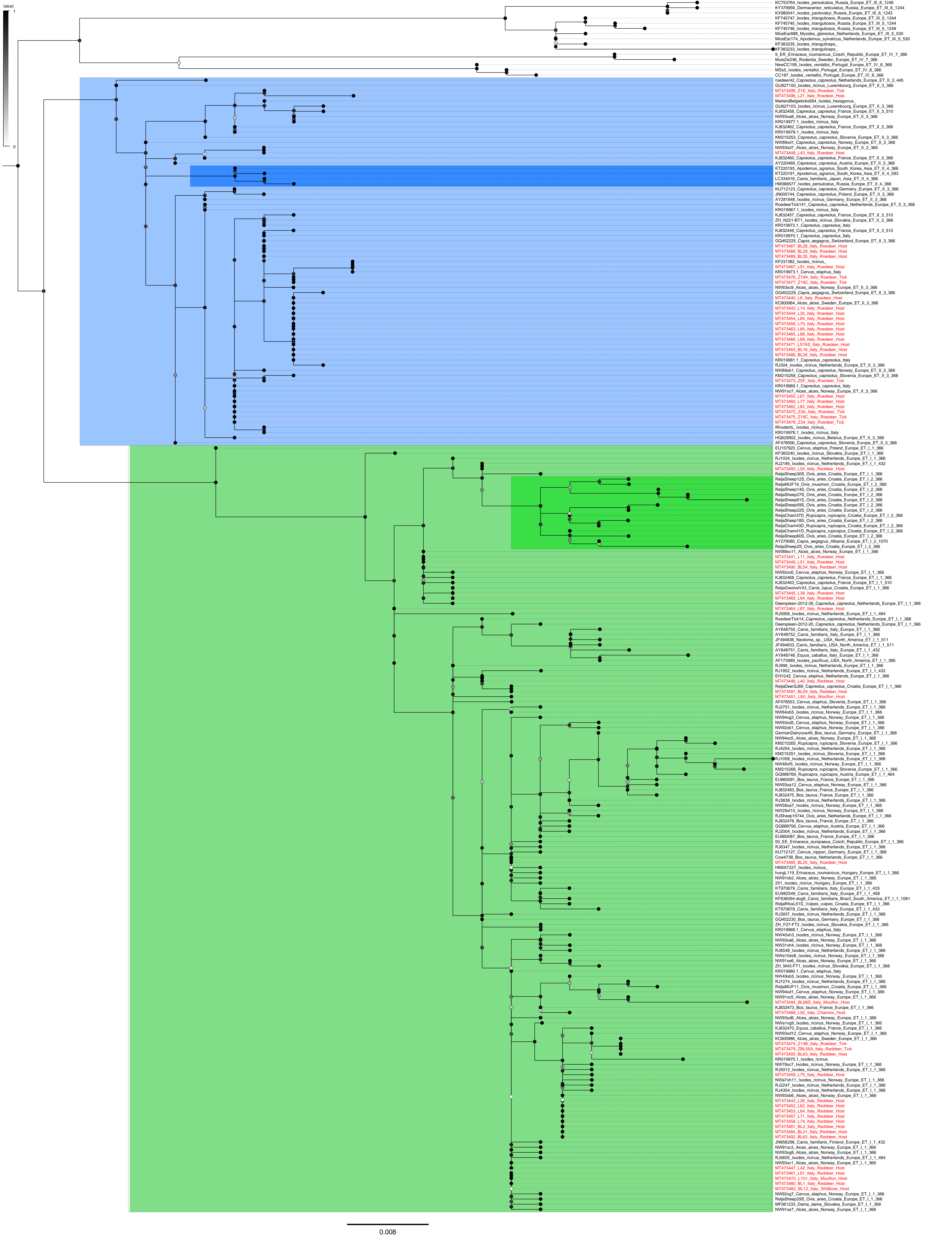

Supplement: Supplementary file 1 [file animals-11-00310-s001.zip › supplementary files/Figure S1 Phylogenetic tree of A. phagocytophilum strains.pdf]
